# Supporting Learner Success: Revisiting Strategic Competence Through Developing an Inventory for Computer-Assisted Speaking Assessment

**DOI:** 10.3389/fpsyg.2021.689581

**Published:** 2021-06-07

**Authors:** Weiwei Zhang, Lawrence Jun Zhang, Aaron John Wilson

**Affiliations:** School of Curriculum and Pedagogy, Faculty of Education and Social Work, The University of Auckland, Auckland, New Zealand

**Keywords:** computer-assisted integrated speaking tests, strategic competence, strategic competence inventory for computer-assisted speaking assessment, English as a foreign/second language, language testing

## Abstract

This study investigated English-as-a-foreign-language (EFL) learners' strategic competence in the computer-assisted integrated speaking tests (CAIST) through the development and validation of the *Strategic Competence Inventory for Computer-assisted Speaking Assessment* (SCICASA). Based on our review of the literature on the CAIST, strategic competence, and available instruments for measuring the construct, we defined EFL learners' strategic competence in the CAIST as learners' use of four metacognitive strategies: Planning, problem-solving, monitoring, and evaluating, with each of them consisting of various components. These metacognitive strategies formulated the four factors and scale items of the SCICASA under validation. An exploratory factor analysis of responses from 254 EFL students and the subsequent confirmatory factor analysis of data collected on another sample of 242 students generated 23 items under the four factors. The high validity and reliability of the SCICASA reveal that EFL learners' strategic competence operates in the forms of the four metacognitive strategies in the CAIST. This will lend some new supporting evidence for Bachman and Palmer's (2010) strategic competence model while providing implications for metacognitive instructions and test development. Concomitantly, the findings show the inventory as a valid instrument for measuring strategic competence in computer-assisted foreign/second language (L2) speaking assessment and relevant research arenas and beyond.

## Introduction

The motivation of this study has to do with one of the authors' teaching experience related to the computer-assisted integrated speaking test (CAIST) in English-as-a-foreign-language (EFL) classrooms. The CAIST measures EFL learners' speaking ability associated with their strategic competence. Such ability is highly valued in tertiary education and is considered as one of the central factors affecting academic success as well as for engaging learners for sustainable growth in language proficiency (Zhang and Zhang, 2019; Teng and Zhang, 2020). Furthermore, the test has been evidenced, though not sufficiently, to elicit strategic competence relevant to tertiary domains (Frost et al., 2020). The close relationship among the CAIST, strategic competence and tertiary education has made the test an effective measurement tool in EFL classroom-based learning (Bahari, 2020). However, when performing the CAIST, students often do not achieve what teachers expect them to achieve, as observed in the classroom teaching. This can be regarded as a concrete example that suggests the necessity of researching EFL learners' strategic competence in the CAIST for helping them achieve academic success (Bachman and Palmer, 2010; Frost et al., 2020).

In actuality, the rapid advance of computer technology and unexpected natural disasters that limit physical contact such as the COVID-19 pandemic have made computer-assisted L2 assessment (CALA) pervasive in L2 learning and teaching at various levels, particularly at the tertiary level (Zhang and Qin, 2018; Qin and Zhang, 2019; Sasere and Makhasane, 2020). As one form of CALA, the CAIST integrates multiple language skills (e.g., reading, listening and speaking) to replicate the authentic language use tasks for evaluating learners' ability to deal with daily language use activities. Such authenticity not only enhances the positive washback effect of the test on classroom-based L2 learning but also improves test fairness, which elucidates the recognition of such a test format as an indicator of the future direction of CALA, and its progressing prominence in high-stakes L2 tests (Bahari, 2020; Frost et al., 2020).

Despite this, as pointed out by some scholars (e.g., Huang and Hung, 2018; Frost et al., 2020), insufficient attention has been devoted to the CAIST, especially EFL learners' strategic competence in the test. In respect to the more general context of the CALA, Winkle and Isbell (2017) commented that the primary focus within CALA is on technological elements, and how strategic competence works in CALA is not clear and needs to be redefined. As the core component of language ability, strategic competence is broadly acknowledged as learners' metacognitive strategy use in L2 assessment, which is well-illustrated in Bachman and Palmer's (2010) strategic competence model (Seong, 2014). According to Bachman (2007), understanding strategic competence is critical to understanding language ability, which is the essence of L2 assessment. Based on this view, research on EFL learners' strategic competence in the CAIST is essentially internal to comprehending the tests *per se*, which will further replenish our apprehension of the CALA, L2 speaking assessment and even L2 assessment at large.

However, although the importance of strategic competence has been recognized across disciplinary boundaries, studies on this construct mainly focus on listening, reading, and writing in non-testing contexts (e.g., Teng and Zhang, 2016, 2020), and hence how strategic competence operates in authentic speaking tests remains unclear (Huang and Hung, 2018; Frost et al., 2020). In addition to the complex nature of strategic competence (Barkaoui et al., 2013), some researchers (e.g., Hughes and Reed, 2017) attributed this research actuality to the complexness of L2 assessment, while the others (e.g., Luoma, 2004; Tarone, 2005) held that as the most difficult language skill for human beings to master, speaking, particularly L2 speaking, is understandably too complex to be researched. The complexity of strategic competence, L2 assessment, speaking, and L2 speech production jointly justify the scant literature regarding the construct in the CAIST on one hand, and the significance of the research attempts that can provide additional evidence for the literature on the other. Given the increasing predomination of CALA in today's educational system and the relationship between strategic competence and the CAIST stated earlier, such research attempts also make great sense to EFL education.

Nevertheless, the research attempts are challenged by the absence of a valid and reliable instrument. To assess individuals' internal strategic processes, including their strategic competence, inventories or questionnaires are regarded as types of effective instruments (Oxford, 2017). Although some inventories are available for investigating learners' strategic competence, they mostly target non-testing contexts (e.g., Oxford, 1990). To our knowledge, inventories that can be employed to examine strategic competence in the CAIST are not yet available. In fact, inventories that can be used in CALA and the more macro L2 speaking assessment are unavailable either. The unavailability has led to the commonly decontextualized use of the accessible strategic competence inventories in empirical studies despite having been criticized by many scholars (e.g., Oxford, 2017; Takeuchi, 2020). Against this background, a valid and reliable inventory is warranted to address the research gap.

Taken together, the scantiness of the exiting literature on researching EFL learners' strategic competence in the CAIST, and the absence of an applicable inventory for such a research attempt indicate the research gaps that the current study is set up to bridge. To this end, we embedded our investigation of strategic competence in the development and validation of a desired inventory in line with some researchers' prior work (e.g., Purpura, 1997; Zhang and Goh, 2006; Teng and Zhang, 2016). Considering the relationship between CALA and the CAIST, our inventory focuses on the more global context of CALA for wider applicability, though our investigation was conducted in the CAIST. For this purpose, we developed and validated the Strategic Competence Inventory for Computer-assisted Speaking Assessment (SCICASA). As our study is the first to integrate research on EFL learners' strategic competence in computer-assisted L2 speaking assessment with instrument development and validation, the uniqueness will provide some new insights into research designs for empirical studies on L2 speaking assessment. Additionally, the findings are expected to offer a valid and reliable inventory for assessing EFL learners' strategic competence in L2 speaking assessment, additional validity evidence for Bachman and Palmer's (2010) strategic competence model, and pedagogic implications for metacognitive scaffolding in EFL classrooms.

## Literature Review

### Computer-Assisted Integrated Speaking Tests

A computer-assisted integrated speaking test (CAIST) is a test format that delivers an integrated speaking test via computer technology. It involves two strands of “young and dynamic” development in L2 assessment: Computer-assisted language assessment and integrated speaking tests (Winkle and Isbell, 2017, p. 313).

Computer-assisted language assessment (CALA), also known as computer-assisted language testing (Pathan, 2012), refers to the use of computer technology for facilitating, contextualizing and enhancing the assessment of test takers' language ability. Concomitant with the speedy and extensive propagation of computer use, CALA has become increasingly common since computers were first employed to score test items in L2 assessment in the 1930's. The growth of CALA has expanded the L2 assessment field and triggered influential washback effects in the L2 classrooms (Winkle and Isbell, 2017). Some researchers (e.g., Booth, 2019) have anticipated CALA as an inevitable and irreversible trend in L2 assessment, which indicates the future of this field due to its advantages, including the individualized test process and simplified test administration. On the other hand, the on-going spread of COVID-19 has further facilitated this trend after online learning and online assessment have been acknowledged as an effective means to normalize the delivery of teaching and learning in challenging situations caused by natural disasters (Sasere and Makhasane, 2020). Based on a review of approximately 300 studies spanning 2002–2018 that examined the mainstream assessment tools in computer-assisted language learning, Bahari (2020) pointed out that CALA is moving toward integrated language skills assessment.

Research efforts focusing on integrated language skills assessment began in the 1970's (Cummings, 2014), but few investigated the integrated speaking tests (Frost et al., 2020). Integrated speaking tests are so called because they integrate reading, listening and speaking to duplicate authentic language use, making it possible to measure learners' ability to communicate in English in real-life settings (Huang and Hung, 2018). It is believed that if learners do well on the tests, they have shown their abilities required in real language use situations where multiple language skills are needed (Luoma, 2004). Built upon the working model of language use in an authentic academic context, integrated speaking tests are theoretically considered as an expanded version of Bachman's (1990) Communicative Language Ability Model. As such, they “broaden the scope of strategies called upon (Barkaoui et al., 2013, p. 16), and are immediately close to the metacognitive strategies of pre-assessment and pre-planning, online planning and monitoring, and post-evaluation (Cohen, 2014). Although the metacognitive strategies assumed to be elicited by integrated speaking tests have not been sufficiently evidenced, as noted earlier, this test format indicates the paramount role of metacognitive strategy use in L2 speech production (Skehan, 2018).

In L2 speech production, monitoring works both covertly and overtly for task completion, and speakers use planning to seek knowledge at hand and monitoring to compensate for, and facilitate, their oral production (Bygate, 2011). In the meanwhile, monitoring operates in conjunction with evaluation (O'Malley and Chamot, 1990; Purpura, 1997), and the speakers have to solve various problems caused by their incomplete L2 knowledge through the use of problem-solving (Kormos, 2011). EFL speakers' metacognitive strategy use in L2 speech production essentially reflects their strategic competence in L2 assessment (Seong, 2014). In other words, the metacognitive strategies that are assumed to be called upon by integrated speaking tests illustrate the equally important part that strategic competence plays in this specific testing context, as it does in L2 assessment (see the subsection of strategic competence). Such importance further warrants a study as the current one.

The delivery of integrated speaking tests by means of CALA is the CAIST, which is typically represented by one of the most influential high-stakes tests: The TOEFL iBt integrated the speaking section (Hughes and Reed, 2017). This explains why existing studies on strategic competence in the CAIST were commonly conducted in the context of the TOEFL iBt (e.g., Barkaoui et al., 2013), which rationalizes the role of this specific test as the research context of our study.

### Strategic Competence

In the research field of L2 assessment, strategic competence is conceived as a set of metacognitive strategies that “provide a management function in language use, as well as in other cognitive activities” (Bachman and Palmer, 2010, p. 48), irrespective of the ambiguity plaguing the conceptualization of the construct (Seong, 2014). Such a conception is due to the profound influence of Bachman and Palmer's (1996; 2010) language ability models, where strategic competence serves as the core component and works independently or interactively with other test factors such as test tasks to considerably influence test performance (Bachman and Palmer, 2010; Skehan, 2018). To illustrate such a core role, several researchers (e.g., Piggin, 2012; Zhang, 2017) regarded strategic competence within the language ability models as an independent model and termed it Bachman and Palmer's strategic competence model, which operates in three forms of metacognitive strategies: Goal setting, appraising, and planning. Goal setting concerns language users' decision on what they seek to do for a given language use task. Appraising helps leaners assess the feasibility of task completion. Planning is about deciding how to use language knowledge for task completion. As a result, researchers typically describe strategic competence as metacognitive strategy use in empirical studies (Seong, 2014).

However, the insufficiency of empirical evidence for the validity of Bachman and Palmer's (2010) strategic competence model makes it hard to define what metacognitive strategies are actually used by learners in real L2 assessment (Ellis et al., 2019). Hence, researchers tended to take an exploratory approach to investigating strategic competence in accordance with the literature on L2 assessment, metacognition, and learning strategies rather than simply defining them as goal setting, appraising, and planning. For example, Barkaoui et al. (2013) discovered that the metacognitive strategies used by Chinese EFL learners were: Identifying the purpose of the task, setting goals, evaluating previous performance, and evaluating the content of what is heard/said. By contrast, in Zhang's (2017) study, the metacognitive strategies that she identified were: Assessing the situation, monitoring, self-evaluation and self-testing. Following these researchers, we defined strategic competence as metacognitive strategy use which was investigated in an exploratory approach. Such an approach is simultaneously consistent with the common practice in inventory development (Creswell and Creswell, 2018).

As an interdisciplinary concept, metacognitive strategies are well-illustrated by the extensively applied three-component model which encompasses planning, monitoring, and evaluating in the research domains of metacognition and language learning strategies (Purpura, 1997; Zhang, 2003; Zhang and Zhang, 2018, 2019). The three components correspond to the constituents of the Bachman and Palmer's (2010) strategic competence model, but they fail to explain problem-solving, the critical strategy in L2 speech production (Bygate, 2011; Kormos, 2011). Additionally, as Seong (2014) commented, derived from Sternberg's (1988) intelligence theory which refers to planning, monitoring and evaluating individuals' problem solving, Bachman and Palmer's (2010) model is considerably influenced by Canale and Swain (1980), who proposed strategic competence as problem-solving mechanisms. Therefore, in the investigation of strategic competence in L2 speaking assessment, it is imperative that problem-solving, side by side with planning, monitoring and evaluating, should be taken into consideration. In light of such imperativeness and guided by an exploratory approach, we adopted Chamot et al.'s (1999). Metacognitive Model of Strategic Learning in formulating the working definitions of the assumed strategic competence elicited by the CAIST.

Comprised of planning, problem-solving, monitoring, and evaluating, Chamot et al.'s (1999) model is built upon interdisciplinary studies on metacognitive strategies involving L2 learners with various backgrounds. It is therefore accepted as empirically grounded (Chamot, 2009). According to Chamot et al. (1999), the inclusion of problem-solving as one component of metacognitive strategies is due to its “usefulness and applicability to a broad range of learning tasks” (p. 11). Moreover, Chamot (2005) pointed out that almost all the models that highlight metacognitive strategies in L2 learning include problem-solving as the fundamental component with planning, monitoring and evaluating (e.g., Chamot et al., 1999; Rubin, 2001; Anderson, 2002). The features of the Chamot et al.'s (1999) model obviously established its correspondence to Bachman and Palmer's (2010) strategic competence model in L2 speaking assessment, but the inclusion of problem-solving makes it better than the latter to theoretically depict the construct in the CAIST. Yet, as Chamot et al.'s model was mainly for non-testing settings, only the components consistent with test contexts are appropriately applicable in the CAIST. In accordance with this, the working definitions and the taxonomies of EFL learners' strategic competence in the CAIST under investigation are formulated in Table 1.

**Table 1:** Definitions and taxonomies of strategic competence in this study.

**MS Taxonomies**		**Definitions**
Planning	Setting goals	Identify the purpose of the task
	Directed attention	Decide in advance to focus on particular tasks and ignore distractions
	Activate background information	Think about and use what you already know to help you do the task
	Prediction	Anticipate information to prepare and give direction for the task
	Organizational planning	Plan the task and content sequence
	Self-management	Arrange for conditions that help you learn
Problem-solving	Inference	Make guesses based on previous knowledge
	Substitute	Use a synonym or descriptive phrase for unknown words
Monitoring	Selective attention	Focus on key words, phrases, and ideas
	Deduction/induction	Consciously apply learned or self-developed rules
	Personalize/personal experience	Relate information to personal experiences
	Take notes	Write down important words and concepts
	Ask if it makes sense	Check understanding and production to keep track of progress and identify problems
	Self-talk	Talk to yourself to reduce anxiety by reminding yourself of progress, resources available, goals
Evaluating	Verify predictions and guesses	Check whether your predictions or guesses are correct
	Check goals	Decide whether a specific goal was met
	Evaluating performance	Judge how well you did in the task

*MS, metacognitive strategies*.

### Measuring Strategic Competence

In empirical studies on strategic competence or metacognitive strategy use, the commonality is that inventories or questionnaires are employed thanks to the properties of the instrument: (a) Easy administration on a large sample size; (b) little intrusiveness; (c) applicability in many statistical analyses; (d) rather high validity and reliability (Craig et al., 2020). In L2 assessment, Purpura's (1997) Metacognitive Strategy Questionnaire (MSQ) has been used extensively for eliciting strategic competence (e.g., Phakiti, 2003, 2008). The 40-item questionnaire has four sections: Assessing the situation, monitoring, self-evaluating and self-testing. A 6-Likert scale ranging from 0 (never) to 5 (always) is used to assess the frequency of the individuals' on-line and off-line metacognitive strategy use in performing reading test tasks. However, the tense and the content of the item questions show that this questionnaire was not designed specifically for L2 assessment. Nevertheless, as this questionnaire was validated by Purpura with structural equation modeling, it has been adapted by several researchers in L2 assessment, including Phakiti (2003), who devised his cognitive and metacognitive questionnaire on EFL reading tests based on the MSQ. Phakiti used fewer items (35 items) and a 5-point Likert scale, which makes his questionnaire more user-friendly. Besides, the past tense and the content in each item have turned the questionnaire into an off-line self-report suitable for the context of reading tests. Later, Phakiti (2008) refined the questionnaire, changing it into an even simpler one with 30 items.

As metacognitive strategies are considered as the subordinate language learning strategies, many questionnaires on this construct are developed in accordance with language learning strategies. One actual instance is Oxford's (1990) Strategy Inventory of Language Learning (SILL), which has been adopted in numerous empirical studies with its high reliability and validity. The SILL is aimed at general learning strategy use, and thus it comprehensively includes six types of strategies: Memory strategies, cognitive strategies, compensation strategies, metacognitive strategies, affective strategies and social strategies with 50 items. Each strategy elicited by one item is measured by its frequency reported on a 5-point Likert scale ranging from 1 (never use it) to 5 (often use it). Though employed widely, for any specific context (e.g., L2 speaking assessment), the SILL is unlikely to be applied directly due to its generalness (Sun et al., 2016).

With regard to L2 speaking, questionnaires that examine metacognitive strategy use in this context are severely lacking. Only one such questionnaire is available: The Metacognitive Awareness Inventory in Listening and Speaking Strategies (MAILSS) developed by Zhang and Goh (2006); see also Zhang (2021). The MAILSS includes 40 items, and the strategies for speaking and listening are categorized into use-focused learning strategies, form-focused learning strategies, comprehension strategies, and communication strategies. The first two strategies are for improving individuals' speaking and listening abilities, while the other two are for enhancing one's comprehension and communicative competence in real-world reciprocal interactions. The use of the metacognitive speaking strategies is rated on a 5-point Likert scale from “Never” (1) to “Very Often” (5). Although the MAILSS can be used to measure metacognitive speaking strategies, it is not developed especially for speaking with its focus on EFL learners' development of metacognitive awareness in non-testing conditions. Because of the limitation, the inventory has not been applied broadly in testing situations (Craig et al., 2020).

From the above exposition, it can be seen that the advantages of questionnaires in assessing metacognitive strategy use rationalizes our development of the SCICASA for investigating EFL learner's strategic competence. In addition, the features of the above four questionnaires, including validity, the participants on whom the instruments are used, the language skills investigated via the instruments, and the contexts (testing or non-testing) where they are applied, account for why we considered these instruments as the original sources of the SCICASA.

## Methods

### SCICASA Development

The development of the SCICASA was essentially a process of narrowing down the strategic competence under investigation. As our research focus was on strategic competence and the research context where the inventory is expected to be applied is computer-assisted L2 speaking assessment, in developing the inventory, we regarded reading, listening, and speaking involved in the CAIST as a macro speaking modality that integrates reading and listening as a prior knowledge provider rather than independent language skills in line with the interpretation of the test format by English Testing Service (ETS) (ETS, 2021a), the developer and organizer of the TOEFL iBt. This indicates that the items in the inventory only relate to speaking, and based on this, we synthesized the scale items in the four questionnaires that suggest EFL learners' metacognitive strategy use in L2 speaking assessment.

Consequently, a total of 40 items that are assumed to elicit the metacognitive strategies and hence to indicate EFL learners' strategic competence in the CAIST were generated, which were classified into planning, problem-solving, monitoring, and evaluating, the four dimensions of the inventory(see Table 1). A sample item on planning was “I knew what the task questions required me to do.” A sample item on problem-solving was “I drew on my background knowledge to complete the task.” Items such as “I knew when I should complete a task more quickly” were used to examine monitoring use and “I evaluated whether my intended plans worked effectively” was one item that investigated the use of evaluating. A 6-point Likert scale was used for each item: 0 (never), 1 (rarely), 2 (sometimes), 3 (often), 4 (usually), and 5 (always). Though the SCICASA was developed in English, each item was operationalised as a written statement in Chinese, the native language of the participants, to reduce possible misunderstandings and enhance the reliability. Five questions on EFL learners' background information such as age and their EFL learning experience were also included in the SCICASA (Sun et al., 2016).

### SCICASA Validation

The validation of the the SCICSA was parsed into two stages: Initial validation relating to its face validity and content validity, and factorial validation focusing on the construct validity and the reliability of the instrument (Byrne, 2016; Kline, 2016). It was in the second stage that we administered the probe into strategic competence in the CAIST.

### Initial Validation

For face and content validity, four PhD students majoring in applied linguistics were consulted on the layout, wording, redundancy, and logic consistency of the inventory. One item that caused misunderstanding was removed. Two Chinese professors with a background of English linguistics were invited to examine the translation of the inventory from original English to Chinese. They scrutinized the items in regard to redundancy, sequencing, clarity, readability, and comprehensibility. Based on their feedback, potentially confusing instructions, interpretations, and the scale items were revised. Modifications were made in item wording, and one new item was added. The modified inventory was then piloted with 22 students to evaluate the wording, the structure and the clarity of the items for the readability and the understandability of the instrument in its actual users (Byrne, 2016). After piloting, the SCICASA (the first draft version) was subject to exploratory factor analysis (EFA) and confirmatory factor analysis (CFA) for its construct validity (Kline, 2016).

### Factorial Validation and Reliability Evaluation

#### Participants

The two factor analyses included data from 496 students based in two universities in a Northern city in the People's Republic of China. The students were recruited via convenience sampling on a voluntary basis, with males and females accounting for 37.64% (*N* = 189) and 63.68% (*N* = 307), respectively. The age range of the participants was between 18 and 21 years, and on average, they reported 10(M = 10.36, SD = 1.95) years of formal English language learning experience.

Almost all the students were enrolled either in the Faculty of Foreign Language Studies or the International Cooperation Programmes in the selected research sites and were in their final academic year before starting their internship or studying abroad related to English, respectively. This English–related background enabled the students to be interested in this study which, they believed, potentially benefited them in their language preparations for their future career or study. Their interest contributed to their cooperation, helping to improve the accuracy of their responses, and hence the validity of the SCICASA was enhanced (Daniel, 2011; Creswell and Creswell, 2018). Additionally, the score range of the students on CET-4, an authoritative test for English language proficiency in China (Zhang, 2017), was from 425 points to 500 points. According to the official scoring interpretation of the test published by the National Education Examinations Authorities (2020), this score range suggests that the students' language proficiency was at an upper-intermediate level as required to take the CAIST (Kyle et al., 2016; Huang and Hung, 2018; Frost et al., 2020).

#### Instruments

To establish a research context of authentic speaking tests, and in line with “cultural neutrality, religious neutrality, and low controversy-provoking possibility” (Huang and Hung, 2013, p. 250), we selected one TOEFL iBt integrated speaking section composed of four tasks from TOEFL practice online data (TPO2). TOEFL practice online tests are official practice tests that feature real past test questions and aim at allowing learners to experience taking the real TOEFL iBt test (ETS, 2021b). Our brief survey showed that none of them had used these practice tests, as they had not been aware of their availability. This ensures the authenticity of the four tasks adopted in our study.

The four speaking tasks involve topics on campus life and academic lectures. The tasks require learners to read and listen or to listen before speaking in response to different task types such as stating an opinion, and arguing for a feasible solution to a problem, during which various amounts of preparation time are provided. We used the test tasks without any changes for authenticity, validity and reliability (Huang and Hung, 2018). It should be noted that the four speaking tasks come from the old version of TOEFL iBt which underwent reform in late 2019.

#### Data Collection

The first cohort of student participants (*N* = 254) was invited to answer the first draft of the SCICASA after they performed the four test tasks in multimedia laboratories. Data collected were used for the EFA, generating the second draft of the SCICASA administered on another different sample of students (*N* = 242) for CFA after they completed the same tasks. To counterbalance the carryover effect, a 20-minute interval between tasks was provided, and the order effect was minimized through a Latin square design (Corriero, 2017). Completing the SCICASA took each student about 20 min, and ethical issues were appropriately addressed after the study was approved by the University of Auckland Human Participants Ethics Committee (Reference Number 020972).

#### Data Analysis

Three steps were involved in EFA: (a) The examination of the feasibility for EFA with reference to Bartlett's test of sphericity (*p* < 0.05) and the Kaiser-Meyer-Olkin (KMO) test (>0.7); (b) factor extraction; and (c) evaluating scale items loading on a particular factor (Byrne, 2016; Kline, 2016). Maximum Likelihood (ML) estimation and Promax rotations were adopted for factor extraction and rotation, respectively (Beavers et al., 2013). In our examination of factor loadings we removed the items that had a factor loading below 0.4 or that loaded on more than one factors from the draft SCICASA (Byrne, 2016; Kline, 2016).

The model extracted through the EFA was then cross-validated in CFA on AMOS 0.24 (Windows version), which started with model specification, model identification, and assumption tests. Model specification was built upon the structure generated from the EFA. Model identification was conducted with reference to the guidelines proposed by Byrne (2016) and Kline (2016), which include: (a) Scaling latent variables (the variance of the first indicator of factors was fixed to a value of 1. 0); (b) deciding on the number of parameters (the number of figures reflected by the input matrix should be not less than the number of freely estimated model parameters); and (c) deciding on the number of indicators of each latent variable (≥ 3). The examination of model fit was based on the fit indices, including Goodness-of-fit (GFI), incremental fit index (IFI), Tucker-Lewis coefficient (TLI), comparative fit index (CFI), and the root-mean-square error approximation (RMSEA). The acceptable cut-off points for GFI, IFI, TLI and CFI were >0.9 and that for RMSEA was <0.8. After factor analyses, the reliability of the inventory was evaluated with reference to the Cronbach's alpha coefficient and the thumb-up criterion was over 0.8. In the CFA, the estimation method of ML was employed as in the EFA (Byrne, 2016; Kline, 2016).

## Results

### Exploratory Factor Analysis

#### Assumption Tests

Descriptive analysis revealed that there were no missing data. Values of the skewness of the items were between −0.018 and 0.427, and the figures for kurtosis ranged from −0.902 to 0.273, all falling within the acceptable bounds for univariate normality. However, 30 multivariate outliers were discovered and removed, making the final sample size to be 224 participants for a 40-item scale, meeting the thumbs-up rule: The subject-to-variable ratio should be 5:1 (Byrne, 2016; Kline, 2016).

The subsequent regression analysis displayed that values of tolerance of the items were all above the cut-off point of 0.2, and the numbers of their variance inflation factor (VIF) were all <5, the cut-off boundary. Such results indicated the absence of multicollinearity. Given the rather large number of items in the SCICSA, linearity was examined between the item with the strong negative skewness and the item with the strong positive skewness via a scatterplot, which also disclosed the multivariate normality. To evaluate the factorability of the dataset, we examined the Bartlett's Test of Sphericity and KMO test via initial factor analysis. The results showed that the strength of the relationships between variables was statistically significant: χ*2* (*df* = 780) = 4740.273, *p* < 0.001, which evidenced that the number of the items (*N* = 40) of the draft SCICASA was statistically sufficient for an EFA procedure (Byrne, 2016; Kline, 2016).

#### Factor Extraction and Rotation

In the initial round of the EFA, with reference to the eigenvalues, the scree plots and the percentage of variance, eight factors were extracted, which explained 62.159 % of the total variance. However, 39 items with their factor loadings above the cut-off value of 0.4 fell on one extracted factor. After factor rotation, numbers from the Pattern Matrix showed that items with factor loadings above 0.4 scattered among the eight factors. Despite this, none of the factors had at least three items (the cut-off criterion), indicating the failure of the factor extraction. Given the parsimony and the meaningfulness of the eight-factor solution in light of the working taxonomies of strategic competence presented in Table 1, an alternative approach to extracting factors was employed in accordance with our review of the relevant literature (Qin, 2003; Byrne, 2016; Kline, 2016) and our consultation with an in-house professor of statistics: The number of factors and their name were determined prior to factor extraction. Accordingly, four factors were generated: Planning, problem-solving, monitoring, and evaluating.

After the first round of EFA on the four-factor solution, the four factors only explained 49.96 of the total variance, and indices of the model fit (GFI) of this solution [χ^2^ (df = 626) = 1105.671, *p* ≤ 0.001] did not demonstrate improvement compared with the eight-factor solution [χ^2^ (*df* = 488) = 700.17, *p* ≤ 0.001]. Meanwhile, values in the Pattern Matrix showed that factor loadings of six items were <0.4 on any of the four factors. After the exclusion of these undesired items in the second round of EFA, a dramatic improvement was seen in the model fit: χ^2^ (*df* = 321) = 590, *p* ≤ 0.001, and the total variance explained by the four factors increased to 54.94%. Following the same procedure, we conducted five rounds of extractions and rotations, which generated a structure composed of 28 items underpinned by the four factors. The proportions of the variance explained by the factors were 39. 23% (planning), 6.66% (monitoring), 6.14% (problem-solving) and 6.14% (evaluating), and the model fit indexes were: χ^2^ (*df* = 272) = 526. 27 (*p* ≤ 0.001), which suggested a good structure. In addition, the output of the Component Correlation Matrix revealed moderate inter factor correlations (≥0.3 but ≤ 0.8), indicating the appropriateness of the Promax rotation run on this dataset (Byrne, 2016; Kline, 2016).

#### Reliability Evaluation

Reliability analysis after the EFA included evaluating the subscale reliability and the full-scale reliability with reference to Cronbach's alpha coefficient α. Results showed that both were above 0.8, revealing good consistency within each factor and within the SCICASA. The factor loadings of the 28 items and their internal and the overall reliability are reported in Table 2.

**Table 2:** Results of EFA and the reliabilities of the four-factor SCICSA.

		**Factor loadings**				
**Factors**	**Items**	***P***	**M**	**PS**	***E***	**α**
P	Q1	0.521				
	Q2	0.513				
	Q3	0.670				
	Q4	0.809				0.886
	Q5	0.734				
	Q6	0.626				
	Q7	0.523				
	Q8	0.683				
	Q9	0.679				
PS	Q14			0.643		
	Q15			0.645		
	Q17			0.740		0.845
	Q19			0.701		
	Q20			0.719		
M	Q23		0.563			
	Q24		0.430			
	Q26		0.473			
	Q27		0.610			0.871
	Q28		0.474			
	Q29		0.787			
	Q30		0.505			
	Q31		0.625			
	Q33		0.628			
E	Q35				0.621	
	Q36				0.595	
	Q37				0.677	0.859
	Q38				0.880	
	Q39				0.654	
Overall reliability		0.941				

*P, planning; PS, problem-solving; M, monitoring; E, evaluating; Q, question; α, Cronbach's alpha*.

### Confirmatory Factor Analysis

#### Model Specification and Model Identification

After model specification and identification, a zero-order model (Model A) composed of four correlated factors was established. In the model, variance of the first indicator of each of the four factors was fixed to 1 by default on the AMOS. Based on the formula of 1/2 [P (P + 1)] where P refers to the number of the items of the SCICASA after EFA (*P* = 28), the number of the parameters in the matrix was 406, greater than that of freely estimated model parameters (62). Moreover, each of the four factors had more than three indicators, the boundary criterion: Nine indicators for planning and monitoring, and five indicators for problem-solving and evaluating. Each indicator was constrained to only one factor with error terms associated with each indicator variable uncorrelated (Byrne, 2016; Kline, 2016).

#### Assumption Tests

In accordance with the cut-off criteria explained above in EFA, values of the skewness (0.051–0.264) and the kurtosis (−0.897 to −0.397) of the 28 items indicated the approximate normal distribution. The subsequent visual inspection of the histograms with normality curves, box plots, and Q-Q plots further evidenced the data normality (Kline, 2016).

In light of the Chi-square value (56.892, α = 0.001, *df* = 28), a total of 24 undesired cases were removed, which reduced the sample size to 218, meeting the suggested requirement: The sample size >200 is considered as a large sample size for CFA. The regression analysis revealed that the values of tolerance were above the cut-off value of 0.2, and the values of their VIF fell within the acceptable boundary (≤ 5), indicating the absence of multicollinearity. Nonetheless, collinearity and homoscedasticity testing showed that there was bivariate non-normality in the variables; hence, the comprehensive multivariate normality was violated (Byrne, 2016; Kline, 2016).

#### Examination of Offending Estimates

Examining the offending estimates was to ensure the feasibility and the statistical significance of all the parameters estimated. It was a fundamental step before model fit evaluation, which included the inspection of the correlation between constructs (convergent validity), standardized factor loadings and standard errors. According to Byrne (2016) and Kline (2016), values of correlation coefficients between constructs should be <0.8; values of standardized factor loadings cannot be close to or exceed 1; and the standard errors should be >0. After the first round of CFA, all these parameters were shown as not offending estimates, though the correlation coefficient between monitoring and evaluating was 0.81, slightly >0.8. Such results suggested the appropriateness of model fit evaluation (Kline, 2016).

#### Model Evaluation

As multivariate normality was violated, multivariate normality was re-investigated during the first round of the CFA. The value of the Mardia's coefficient multivariate kurtosis was found to be 136.091, and its critical ratio or C.R. was 24.286, both greater than the threshold criteria: Normalized multivariate kurtosis should be <5, and the value of C.R. should be <1.96. Therefore, multivariate non-normality was identified. For non-normal correction, bootstrapping procedure was run so that the bias-corrected confidence intervals of the parameter estimate, and the corrected general model fit indices were examined for model evaluation (Byrne, 2016; Kline, 2016).

Results of the model fit indices of Model A were: χ^2^ (*df* = 344) = 750.034, *p* = 0.000. As the value of χ^2^/*df* was 2.18, larger than the cut-off point (≥ 2), and the *p-*value was found to be 0.00, less than the thumb-up value of 0.05, the model was not satisfactory. Additionally, values of CFI, GFI, and TLI were all <0.9, the criteria for an acceptable model. Given that these indices were estimated under the condition of multivariate non-normality, bootstrap standard errors of each parameters and bootstrap confidence were inspected for bias corrected parameters. After the bias correction, all these indices were statistically significant: *p-*values of the bootstrap standard errors were <0.001, while their bootstrap confidence did not fall on the value of zero. Bollen-Stine bootstrap value was also examined for the bias-corrected general model fit which was equal to zero. The outcome of the bootstrapping was consistent with the original model fit examination, suggesting that Model A did not fit the current dataset and therefore modification was needed for a better model fit (Byrne, 2016; Kline, 2016).

#### Model Modifications

**Figure 1:**
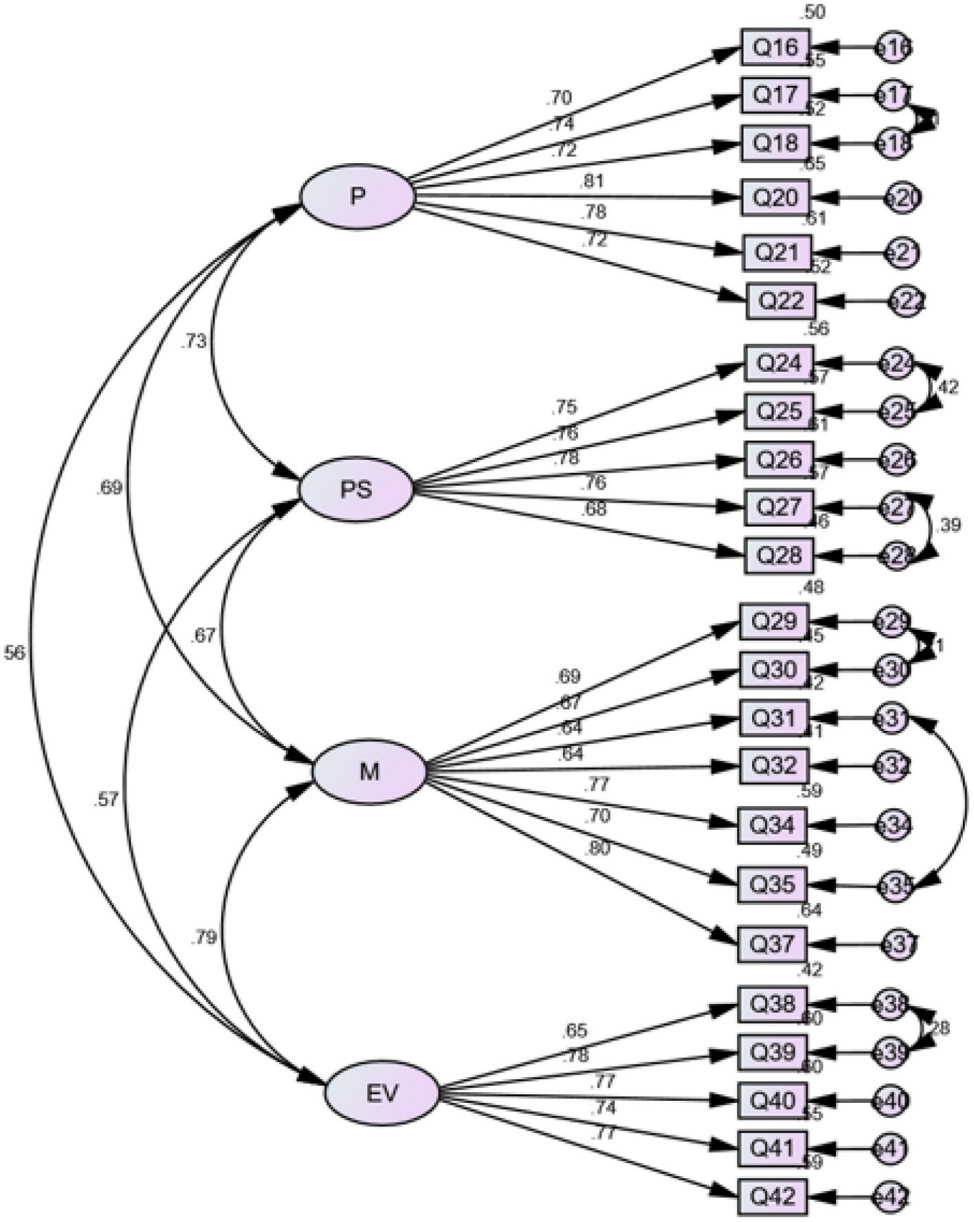
Model D. P, planning; PS, problem-solving; M, monitoring; EV, evaluation; Q, Question.

Model modification was conducted with reference to factor loadings, modification indices and standardized residual weights. As Byrne (2016) and Kline (2016) proposed, an ideal factor loading should be >0.7. Further, the observed variables with standardized residual weight >1.6 for *p* < 0.05 may indicate areas of strain and should be removed. In line with this, two undesired items were deleted, which improved the model fit and generated Model B. The inspection of the modification indices of Model B led to the inclusion of extra six paths between error terms, which resulted in a better Model C. Final modification involved the deletion of variables with undesired standardized residual weights. After the modification, Model D composed of 23 items was established with desired model fit indices: Although the index of CFI (0.892) was still less than the cut-off value of 0.9, other indices were satisfactory. In addition, the bootstrap estimates proved that the bias-corrected bootstrap standard errors and the intervals of the parameters in the model were all acceptable. In addition, a bias-corrected *p*-value of Model D was 0.204, much greater than the threshold (0.05), indicating the statistical significance of the model fit. Detailed model indices of the four models generated in CFA are summarized in Table 3, and Figure 1 illustrates the factor loadings of the 23 items and the correlation coefficients of the four factors in Model D.

**Table 3:** Model fit indices for four rounds of modifications.

**Models**	**χ^**2**^**	**CMIN/DF**	**CFI**	**GFI**	**TLI**	**RMESEA**	**SRMR**
Model A	750.034	2.18	0.884	0.799	0.872	0.074	0.0616
Model B	628.765	2.14	0.897	0.814	0.886	0.073	0.0608
Model C	553.975	1.93	0.919	0.839	0.908	0.066	0.0567
Model D	302.577	1.388	0.968	0.892	0.963	0.043	0.0512

#### Validity and Reliability

Model validity and reliability were assessed with reference to the values of the Composite Reliability (CR > 0.7), Average Variance Extracted (AVE > 0.5) and Maximum Shared Variance (MSV ≤ AVE). As shown in Table 4, the values of CR for the factors were satisfactory; the value of AVE for monitoring was a little less than the cut-off criterion of 0.5; and the values of MSV for monitoring and evaluation were slightly greater than the values of their AVE. Those numbers indicated that Model D did not meet the requirements on construct validity. Despite this, given the fairly large sample size, and the ideal overall fit indices demonstrated by Table 3, this minor discrepancy between the actual values and the cut-off points was tolerable (Byrne, 2016; Kline, 2016). Therefore, Model D was accepted to fit the dataset.

**Table 4:** Validity and reliability of Model D.

**Factors**	**CR**	**AVE**	**MSV**
M	0.893	0.483	0.664
P	0.910	0.558	0.452
PS	0.878	0.591	0.436
E	0.867	0.566	0.664

*M, monitoring; P, planning; PS, problem-solving; E, evaluating*.

## Discussion

The development and validation of the SCICASA was essentially a process in which we probed the EFL learners' strategic competence in the CAIST. The 4-factor model validated via EFA and CFA (see Figure 1) revealed EFL learners' strategic competence in the CAIST. To be specific, the first factor, linguistically labeled planning, refers to EFL learners' determination of their objectives and how to achieve the expected goals in test performance. This planning construct is reflected by six items (e.g., I was aware of the need to plan a course of action). The second factor is labeled problem-solving, which is highly related to what the EFL learners did when encountering problems in the test such as making a guess or using a substitute. Five items represent this factor and examples of these items include “I guessed the meaning of the unknown words or expressions by using my knowledge (e.g., words in the context, knowledge of word information and of the topic).” The third construct is monitoring which refers to the EFL learners' examination of what they did in the test for a given plan. An example item is “When I was speaking, I knew when I had spoken in a way that sounded like a native speaker.” The fourth factor is evaluating, which displayed the EFL students' response to post-test self-evaluation. The construct is represented by items such as “I evaluated my performance satisfaction as I moved along the task.” Detailed constructs and the item scales of the SCICASA that reflect EFL learners' strategic competence in the CAIST are presented in the Appendix.

In addition, the SCICASA helped us identify that problem-solving, though typically not included in the widely applied three-component model of metacognitive strategies, is one of the fundamental components of EFL learners' strategic competence in the CAIST. Given that the participants are Chinese EFL learners, such identification is supported by studies such as Sun (2016) and Zhou (2020), who postulated that problem-solving is one of the key strategies that Chinese EFL learners must master in their daily EFL learning activities. Learning experiences of such likely made it natural for the participants to use problem-solving in L2 assessment. Chinese EFL learners' problem-solving strategy use as evidenced in the inventory was also reported in the study by Yin (2013), who discovered that problem-solving worked more effectively in Chinese EFL learners' performance on speaking tasks. In fact, this phenomenon is not unique to Chinese EFL learners. According to Cohen (2018), L2 learners tend to use strategies in line with a specific language skill or modality, which put strategy use in a well-placed position. Additionally, such a relationship coincides with the view held by Oxford (2017), who proposed that the use of L2 learning strategies, including metacognitive strategies, is associated with a specific language skill area.

Furthermore, the validation provides empirical support for some researchers who advocated the inclusion of problem-solving in the metacognition model (e.g., Chamot, 2009). It accordingly proves that Bachman and Palmer's (2010) strategic competence model should be reconsidered, and the problem-solving strategy is expected to be included. The inclusion will serve as a research effort to respond to the proposal from some scholars in L2 assessment: Metacognitive strategies validated by empirical studies should be included in Bachman and Palmer's (2010) strategic competence model for its comprehensive validity (e.g., Phakiti, 2016). On the other hand, the validation of planning, monitoring, and evaluating reflected in the SCICASA lends support for Bachman and Palmer's (2010) model. Moreover, the high correlation coefficients of the four constructs validated by the CFA demonstrate the interactions of the four individual metacognitive strategies with one another in the EFL learners' response to L2 speaking assessment. The interactions are consistent with the working mode of the construct advocated by scholars such as Flavell (1979) and Takeuchi (2020): Metacognitive strategies operate either independently or interactively in task performance. Finally, from the perspective of speaking, the inventory adds validating evidence for the L2 speech production models proposed by Kormos (2011) and Bygate (2011), where planning, problem-solving, monitoring, and evaluating work independently and interactively.

## Conclusion and Implications

We conducted our investigation into EFL learners' strategic competence in the CAIST through developing and validating the SCICASA. The high validity and reliability of the inventory reveal that in performing the test, EFL learners used planning, problem-solving, monitoring, and evaluating as assumed. Our pioneering attempt to integrate our study on strategic competence in L2 speaking assessment into instrument development and validation will provide some new insights into research design for researchers in this field. Given the decontextualization of the available metacognitive strategy questionnaires in the field, the presence of the SCICASA may help address, to some degree, this problem in empirical studies. In addition, the availability of the inventory will permit language educators to understand learners' internal strategic response to L2 integrated speaking test tasks. Considering the extensively recognized washback effect of the test format, such an understanding will provide pedagogical implications for EFL teachers' classroom instructions related to metacognitive scaffolding, especially in teaching EFL learners in China, a context where English is not widely used as a common lingua franca. In the Chinese context, research on strategic competence mainly focuses on the teachability of the construct, and little is known on how Chinese EFL learners' strategic competence works in actual learning activities (Wang et al., 2015). Similarly, the new instrument might have a role in helping test developers examine whether test tasks truly elicit assumed strategic behaviors from test-takers as required for meeting the assumptions of test validity and reliability (Bachman and Palmer, 2010). Furthermore, the use of the SCICASA may help redefine strategic competence in CALA for the advancement of such a cutting-edge format in L2 assessment advocated by some researchers (e.g., Park, 2018).

Of note is that, although the context where the SCICASA was custom-designed is the CAIST, the contextualisation does not exclude the employment of the inventory in speaking tests in any forms and the speaking activities in non-testing contexts. The reason has to do with the diverse sources from which the inventory was developed.

## Limitations and Further Steps

Due to the convenience sampling, the participants had similar backgrounds, particularly, in their English learning experience and the level of EFL proficiency. Additionally, a total of 496 participants might meet the sample size requirement for EFA and CFA (e.g., Byrne, 2016), but may not reach the thump-up criterion expected by others. As a result, the limitations caused by the participants' homogeneity and the sample size may restrict the generalisability of the research results to other populations (Gurven, 2018). Further, although the SCICASA is expected to be used in the computer-assisted L2 speaking assessment, the scale items primarily focus on strategic competence and hence do not reflect the properties of the computer-assisted testing, which may weaken the contextualization of the inventory.

It is therefore necessary for us to recommend that a larger sample size characterized by more heterogeneity be adopted in future research so that the representativeness of EFL learners will be enhanced. In addition, the items pertaining to the characteristics of the computer-assisted assessment context, such as EFL learners' familiarity with the testing equipment that may influence their strategy use, should be included in the inventories of relevance for better contextualisation.

## Data Availability Statement

The original contributions presented in the study are included in the article/supplementary material, further inquiries can be directed to the corresponding author/s.

## Ethics Statement

The studies involving human participants were reviewed and approved by The University of Auckland Human Participants Ethics Committee. The patients/participants provided their written informed consent to participate in this study.

## Author Contributions

WZ conceived of the initial idea, fine-tuned by LZ and AW. WZ designed the study, collected, analyzed the data, and drafted the manuscript. LZ and AW revised and proofread the manuscript. LZ finalized and submitted it as the corresponding author. All authors contributed to the article and approved the submitted version.

## Conflict of Interest

The authors declare that the research was conducted in the absence of any commercial or financial relationships that could be construed as a potential conflict of interest.

## Appendix

### The Strategic Competence Inventory for Computer-Assisted Speaking Assessment

#### Part One

Please provide your information by ticking (√) in the box or write your responses in the space so we can better understand your answers.

- Code:
- Age:
- Gender: Male_□ Female_□ Gender diverse_□
- The years you have been learning English to present:

7~9 years_ □ 10~12 years_□ 13~15 years_□ Others_______.

- English proficiency reflected by test

CET4_□ CET6_□_ BEC_□ IELTS_□ TOEFL_□

#### Part Two

Please read each of the following statements and indicate how you thought about completing the task during the integrated speaking test by ticking (√) 0 (never), 1 (rarely), 2 (sometimes), 3 (often), 4 (usually), and 5 (always)

**Table:**

**Your thinking**	**0**	**1**	**2**	**3**	**4**	**5**
1. I knew what the task questions required me to do						
2. I was aware of the need to plan a course of action.						
3. I thought about what to do to complete the task well						
4. I made sure I clarified the goals of the task						
5. I understood the essential steps needed to complete the task						
6. I organized the structure of what I was going to say before speaking.						
7. I guessed the meaning of the unknown words or expressions by using my knowledge (e.g., words in the context, knowledge of word information, knowledge of the topic.						
8. I used the context to guess the topic						
9. I drew on my background knowledge to complete the task						
10. I made up new words or guess if I didn't know the right ones to use						
11. I used a word or phrase that means the same thing when I could not think of a word in English.						
12. I knew when I should complete a task more quickly						
13. I knew when I should complete a task more carefully						
14. I knew how much time had gone by.						
15. When I was speaking, I knew when I had spoken in a way that sounded like a native speaker.						
16. I related the incoming information to what I had known						
17. When I was performing my task, I took notes on the important words and concepts.						
18. I knew what to do if my intended plan did not work efficiently during the task.						
19. I mentally give myself a grade after I finished my task						
20. I checked whether I had accomplished my goal after completing my task.						
21. I checked the mistakes I had made in the task.						
22. I evaluated my performance satisfaction as I moved along the task.						
23. I evaluated whether my intended plans worked effectively						

## References

[B1] Anderson N. J. (2002). The Role of Metacognition in Second Language Teaching and Learning. ERIC ED463659. Available online at: https://eric.ed.gov/?id=ED463659

[B2] Bachman L. F. (1990). Fundamental Considerations in Language Testing. Oxford: Oxford University Press.

[B3] Bachman L. F. (2007). What is the construct? The dialectic of abilities and contexts in defining constructs in language assessment, in Language Testing Reconsidered, eds Fox J. Wesche M. Bayliss D. Cheng L. Turner C. E. Doe C. (Ottawa, ON: University of Ottawa Press), 41–71. 10.2307/j.ctt1ckpccf.9

[B4] Bachman L. F. Palmer A. S. (1996). Language Testing in Practice: Designing and Developing Useful Language Tests. Oxford: Oxford University Press.

[B5] Bachman L. F. Palmer A. S. (2010). Language Assessment in Practice: Developing Language Assessments and Justifying Their Use in the Real World. Oxford: Oxford University Press.

[B6] Bahari A. (2020). Computer-assisted language proficiency assessment tools and strategies. Open Learn. 36, 61–87. 10.1080/02680513.2020.1814229

[B7] Barkaoui K. Brooks L. Swain M. Lapkin S. (2013). Test-takers' strategic behaviours in independent and integrated speaking tasks. Appl. Linguist. 34, 304–324. 10.1093/applin/ams046

[B8] Beavers A. S. Lounsbury J. W. Richards J. K. Huck S. W. Skolits G. J. Esquivel S. L. (2013). Practical considerations for using exploratory factor analysis in educational research. Pract. Assessment Res. Evaluat. 18, 1–13. 10.7275/qv2q-rk76

[B9] Booth D. (2019). Computer-Based Language Assessment: The Future is Here. Available online at: https://www.english.com/blog/computer-based-language-assessment/ (accessed May 9, 2021).

[B10] Bygate M. (2011). Teaching and testing speaking, in The Handbook of Language Teaching, eds Long M. H. Doughty C. J. (West Sussex: Wiley), 412–440. 10.1002/9781444315783.ch23

[B11] Byrne B. M. (2016). Structural Equation Modelling With AMOS Basic Concepts, Applications, and Program (3rd ed.). New York, NY: Routledge. 10.4324/9781315757421

[B12] Canale M. Swain M. (1980). Theoretical bases of communicative approaches to second language teaching and testing. Appl. Linguist. 1, 1–47. 10.1093/applin/1.1.1

[B13] Chamot A. Barnhardt S. Beard El- Dinary P. Robbins J. (1999). The Learning Strategy Handbook. New York, NY: Longman.

[B14] Chamot A. U. (2005). Language learning strategy instruction: current issues and research. Ann. Rev. Appl. Linguist. 25, 112–130. 10.1017/S0267190505000061

[B15] Chamot A. U. (2009). The CALLA Handbook: Implementing the Cognitive Academic Language Learning Approach (2nd ed.). White Plains, NY: Pearson Education.

[B16] Cohen A. D. (2014). Strategies in Learning and Using a Second Language. London: Routledge. 10.4324/9781315833200

[B17] Cohen A. D. (2018). Moving from theory to practice: a close look at language learner strategies, in Language Learning Strategies and Individual Learner Characteristics: Situating Strategy Use in Diverse Contexts, eds Oxford R. L. Amerstorfer C. M. (London: Bloomsbury).

[B18] Corriero E. F. (2017). Counterbalancing, in The Sage Encyclopaedia of Communication Research Methods, ed Allen M. (Los Angeles, CA: Sage).

[B19] Craig K. Hale D. Grainger C. Stewart M. E. (2020). Evaluating metacognitive self-reports: systematic reviews of the value of self-report in metacognitive research. Metacogn. Learn. 15, 155–213. 10.1007/s11409-020-09222-y

[B20] Creswell J. W. Creswell J. D. (2018). Research Design: Qualitative, Quantitative, and Mixed Methods Approaches (5th ed.). Los Angeles, CA: Sage.

[B21] Cummings A. (2014). Assessing integrated skills, in The Companion to Language Assessment, ed Kunnan A. J. (New York, NY: Wiley), 1–13.

[B22] Daniel J. N. (2011). Sampling Essentials: Practical Guidelines for Making Sampling Choices. Los Angeles, CA: Sage. 10.4135/9781452272047

[B23] Ellis R. Skehan P. Li S. Shintani N. Lambert C. (2019). Task-Based Language Teaching: Theory and Practice. Cambridge: Cambridge University Press. 10.1017/9781108643689

[B24] ETS (2021a). TOEFL iBT^®^ Speaking Section: What is in the Speaking Section? Available online at: https://www.ets.org/toefl/test-takers/ibt/prepare/tests/ (accessed May 9, 2021).

[B25] ETS (2021b). TOEFL iBT^®^ Practice Tests: TOEFL iBT^®^ Practice Sets. Available online at: https://www.ets.org/toefl/test-takers/ibt/prepare/tests/ (accessed May 9, 2021).

[B26] Flavell J. H. (1979). Metacognition and cognitive monitoring: a new area of cognitive-developmental inquiry. Am. Psychol. 34, 906–911. 10.1037/0003-066X.34.10.906

[B27] Frost K. Clothier J. Huisman A. Wigglesworth G. (2020). Responding to a TOEFL iBT integrated speaking task: mapping task demands and test takers' use of stimulus content. Language Testing, 37, 133–155. 10.1177/0265532219860750

[B28] Gurven M. (2018). Broadening horizons: sample diversity and socioecological theory are essential to the future of psychological science. Proc. Natl. Acad. Sci. U.S.A. 115, 11420–11427. 10.1073/pnas.1720433115 30397108 PMC6233064

[B29] Huang H. D. Hung S. T. A. (2013). Comparing the effects of test anxiety on independent and integrated speaking test performance. TESOL Q. 47, 244–269. 10.1002/tesq.69

[B30] Huang H. T. D. Hung S. T. A. (2018). Investigating the strategic behaviours in integrated speaking assessment. System 78, 201–212. 10.1016/j.system.2018.09.007

[B31] Hughes R. Reed B. S. (2017). Teaching and Researching Speaking (3rd ed.). New York, NY: Routledge. 10.4324/9781315692395

[B32] Kline R. B. (2016). Principles and Practice of Structural Equation Modelling (4th ed.). New York, NY: Guilford.

[B33] Kormos J. (2011). Speech production and the cognition hypothesis, in Second *Language Task Complexity: Researching the Cognition Hypothesis of Language Learning and Performance*, ed Robinson P. (Amsterdam: Benjamins). 10.1075/tblt.2.06ch2

[B34] Kyle K. Crossley S. A. Mcnamara D. S. (2016). Construct validity in TOEFL iBT speaking tasks: insights from natural language processing. Lang. Testing 33, 319–340. 10.1177/0265532215587391

[B35] Luoma S. (2004). Assessing Speaking. Cambridge: Cambridge University Press. 10.1017/CBO9780511733017

[B36] National Education Examinations Authorities (2020). How to Interpret Scores for the College English Test. Beijing, China: Ministry of Education of China.

[B37] O'Malley J. M. Chamot A. U. (1990). Learning Strategies in Second Language Acquisition. Cambridge: Cambridge University Press. 10.1017/CBO9781139524490

[B38] Oxford R. L. (1990). Language Learning Strategies: What Every Teacher Should Know. Boston, MA: Heinle & Heinle.

[B39] Oxford R. L. (2017). Teaching and Researching Language Learning Strategies. New York, NY: Routledge. 10.4324/9781315719146

[B40] Park M. (2018). Innovative assessment of aviation English in a virtual world: Windows into cognitive and metacognitive strategies. ReCALL: J. EUROCALL 30, 196–123. 10.1017/S0958344017000362

[B41] Pathan M. M. (2012). Computer Assisted Language Testing [CALT]: advantages, implications and limitations. Research Vistas, 1, 30–45.

[B42] Phakiti A. (2003). A closer look at the relationship of cognitive and metacognitive strategy use to EFL reading achievement test performance. Lang. Test. 20, 26–56. 10.1191/0265532203lt243oa

[B43] Phakiti A. (2008). Construct validation of Bachman and Palmer's 1996 strategic competence model over time in EFL reading tests. Lang. Test. 25, 237–272. 10.1177/0265532207086783

[B44] Phakiti A. (2016). Test-takers' performance appraisals, appraisal calibration, and cognitive and metacognitive strategy use. Lang. Assess. Q. 13, 75–108. 10.1080/15434303.2016.1154555

[B45] Piggin G. (2012). What are our tools really made out of? A critical assessment of recent models of language proficiency. Polyglossia 22, 79–87.

[B46] Purpura J. E. (1997). An analysis of the relationships between test-takers' cognitive and metacognitive strategy use and second language test performance. Lang. Learn. 47, 289–325. 10.1111/0023-8333.91997009

[B47] Qin T. L. Zhang L. J. (2019). English as a foreign language writers' metacognitive strategy knowledge of writing and their writing performance in multimedia environments. J. Writing Res. 11, 393–413. 10.17239/jowr-2019.11.02.06

[B48] QinX. (2003). 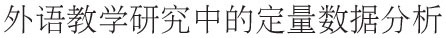 [Quantitative Data Analysis in Foreign Language Teaching and Research]. Wuhan: HUST Press.

[B49] Rubin J. (2001). Language learner self-management. J. Asian Pac. Commun. 11, 25–37. 10.1075/japc.11.1.05rub

[B50] Sasere O. B. Makhasane S. D. (2020). Global perceptions of faculties on virtual programme delivery and assessment in higher education institutions during the 2020 covid-19 pandemic. Int. J. Higher Educ. 9, 181–192. 10.5430/ijhe.v9n5p181

[B51] Seong Y. (2014). Strategic competence and L2 speaking assessment. Teachers College, Columbia University Working Papers in TESOL & Applied Linguistics.

[B52] Skehan P. (2018). Second *Language Task-Based Performance: Theory, Research, Assessment*. New York, NY: Routledge. 10.4324/9781315629766

[B53] Sternberg R. J. (1988). The Triarchic Mind: A New Theory of Human Intelligence. New York, NY: Viking.

[B54] Sun P. P. (2016). Chinese as a second language learners' speech competence and speech performance in classroom contexts: cognitive, affective, and socio-cultural perspectives (Unpublished Ph.D. thesis), The University of Auckland, Auckland.

[B55] Sun P. P. Zhang L. Gray S. (2016). Development and validation of the speaking strategy inventory for learners of Chinese (SSILC) as a second/foreign language. Asia-Pacific Educ. Res. 25, 593–604. 10.1007/s40299-016-0287-0

[B56] Takeuchi O. (2020). Language learning strategies: insights from the past and directions for the future, in Second *Handbook of English Language Teaching*, ed Gao X. (Cham: Springer).

[B57] Tarone E. (2005). Speaking in a second language, in Handbook of Research in Second Language Teaching and Learning, ed Hinkel E. (Mahwah, NJ: Erlbaum).

[B58] Teng L. S. Zhang L. J. (2016). A Questionnaire-based validation of multidimensional models of self-regulated learning strategies. Modern Lang. J. 100, 674–701. 10.1111/modl.12339

[B59] Teng L. S. Zhang L. J. (2020). Empowering learners in the second/foreign language classroom: can self-regulated learning strategies-based writing instruction make a difference? J. Second Lang. Writing 48, 1–12. 10.1016/j.jslw.2019.100701

[B60] Wang D. Lai H. Leslie M. (2015). Chinese English learners' strategic competence. J. Psycholinguist. Res. 44, 701–714. 10.1007/s10936-014-9313-7 25134668

[B61] Winkle P. M. Isbell D. B. (2017). Computer-assisted language assessment, in Language, Education and Technology, eds Thorne S. L. May S. (New York, NY: Springer), 313–323. 10.1007/978-3-319-02237-6_25

[B62] Yin Z. (2013). Infer the meaning of unknown words by sheer guess or by clues? An exploration on the clue use in Chinese EFL learner's lexical inferencing. Engl. Lang. Teach. 6, 29–38. 10.5539/elt.v6n11p29

[B63] Zhang D. Goh C. C. M. (2006). Strategy knowledge and perceived strategy use: Singaporean students' awareness of listening and speaking strategies. Lang. Awareness 15, 199–119. 10.2167/la342.0

[B64] Zhang D. Zhang L. J. (2019). Metacognition and self-regulated learning (SRL) in second/foreign language teaching, in Second *Handbook of English Language Teaching*, ed Gao X. (Cham: Springer). 10.1007/978-3-030-02899-2_47

[B65] Zhang L. (2017). Metacognitive and Cognitive Strategy Use in Reading Comprehension: A Structural Equation Modelling Approach. Singapore: Springer. 10.1007/978-981-10-6325-1_5

[B66] Zhang L. J. (2003). Research into Chinese EFL learner strategies: Methods, findings and instructional issues. RELC J. 34, 284–322. 10.1177/003368820303400303

[B67] Zhang L. J. Qin T. L. (2018). Validating a questionnaire on EFL writers' metacognitive awareness of writing strategies in multimedia environments, in Metacognition in Language Learning and Teaching, eds Haukås A. Bjørke C. Dypedahl M. (London: Routledge), 157–179. 10.4324/9781351049146-9

[B68] Zhang L. J. Zhang D. (2018). Metacognition in TESOL: theory and practice, in The TESOL Encyclopaedia of English Language Teaching, Vol. II, eds Liontas J. I. Shehadeh A. (Hoboken, NJ: Wiley-Blackwell), 682–792. 10.1002/9781118784235.eelt0803

[B69] Zhang W. W. (2021). Strategic competence, task complexity, and learner performance in computer-delivered integrated speaking test tasks: a study of English-as-a-Foreign-Language (EFL) learners in China (Unpublished Ph.D. thesis), The University of Auckland, Auckland.

[B70] ZhouL. (2020). 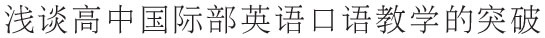 [On how to make a breakthrough in teaching Chinese high school EFL learners speaking skill in international schools]. 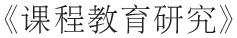 Curriculum Edu. Res. 8, 112–113. Available online at: https://www.doc88.com/p-91773113656751.html?r=1

